# Choroidal Vascularity Index in Central and Branch Retinal Vein Occlusion

**DOI:** 10.3390/jcm11164756

**Published:** 2022-08-15

**Authors:** Pasquale Loiudice, Giuseppe Covello, Michele Figus, Chiara Posarelli, Maria Sole Sartini, Giamberto Casini

**Affiliations:** 1 Department of Surgical, Medical and Molecular Pathology and Critical Care Medicine, University of Pisa, 56124 Pisa, Italy; 2 Complex Operative Ophthalmology Unit, “F. Lotti” Hospital, 56025 Pontedera, Italy

**Keywords:** branch vein occlusion, central vein occlusion, choroid, choroidal vascularity index, image binarization, macular oedema, optical coherence tomography, retinal vein occlusion

## Abstract

(1) Background: we aimed to evaluate choroidal vascularity change in eyes with central and branch retinal vein occlusion (RVO). (2) Methods: in this retrospective cross-sectional study, we reviewed the records of 47 patients with recent-onset, naïve, unilateral retinal vein occlusion. Enhanced-depth imaging optical coherence tomography scans were binarized using the ImageJ software; luminal area (LA) and total choroidal area (TCA) were measured. The choroidal vascularity index (CVI) was calculated as the proportion of LA to TCA. Depending on the pattern of macular oedema, eyes were classified as having no macular oedema (nME), cystoid macular oedema (CME), cystoid macular oedema with serous retinal detachment (mixed). (3) Results: CVI, TCA and LA were greater in eyes with RVO than in fellow, unaffected eyes. No difference was found between central and branch RVO except for central macular thickness (CMT). When compared with controls, eyes with CME presented a significant increase in subfoveal choroidal thickness, CMT, TCA, LA and CVI; eyes with mixed macular oedema had greater CMT and CVI than contralateral eyes; no significant differences in any of the considered parameters were observed in eyes with nME. (4) Conclusions: The results suggest that RVO alters the vascularity of the choroid that varies according to the type of macular oedema.

## 1. Introduction

Retinal vein occlusion (RVO) is an expression used to cover a spectrum of conditions characterized by impaired retinal venous flow. RVOs can be classified as central retinal vein occlusion (CRVO), hemi-retinal vein occlusion (HRVO) and branch retinal vein occlusion (BRVO), depending on the location of the obstruction. Taken together, they represent the second leading cause of blindness due to retinal vascular disorders after diabetic retinopathy [1]. Symptomatology can range from nearly asymptomatic to impaired light perception due to macular edema and ischemia, optic neuropathy, vitreous hemorrhage, neovascular glaucoma, proliferative retinopathy, tractional or combined tractional/rhegmatogenous retinal detachment [2,3].

Since the use of enhanced deep image optical coherence tomography (EDI-OCT) has allowed better visualization of the choroid [4], several authors have studied choroidal change in eyes with CRVO or BRVO leading to discordant results. On the one hand, an increase in subfoveal choroidal thickness was found (SFCT) in patients with RVO if compared with unaffected fellow eyes [5,6,7]; on the other hand, other authors did not find any significant difference in SFCT between eyes with RVO and contralateral ones [8,9].

Derived from the research of Sonoda and colleagues [10] and further developed by Agrawal and co-workers [11], the choroidal vascularity index (CVI) has been considered a promising tool to evaluate choroidal vascular change since it is not influenced by age, gender, refractive error, axial length or intraocular pressure. Defined as the proportion of luminal area (LA) to total choroidal area (TCA), it has been employed in several ocular diseases such as central serous chorioretinopathy, age-related macular degeneration, type-2 diabetes and Vogt–Koyanagi–Harada Disease [12,13,14,15].

The present study aimed to evaluate if, in eyes with recent-onset, naïve, unilateral retinal vein occlusion, (1) CRVO and BRVO induce modification in choroidal vascularity assessed by the CVI, (2) there is any difference in CVI between central and branch RVO, (3) there is a relationship between the type of macular edema and choroidal response to retinal vein occlusion. 

## 2. Materials and Methods

This retrospective cross-sectional study was approved by the local Institutional Review Board (Comitato Etico Area Vasta Nord-Ovest, Prot. n. 17441) of Pisa University Hospital, Pisa, Italy. The study was conducted in adherence with the tenets of the current version of the Declaration of Helsinki (64th WMA General Assembly, Fortaleza, Brazil, October 2013). All patients signed an informed consent form.

We reviewed the medical records of patients with recent-onset, naïve, unilateral retinal vein occlusion who were referred to Pisa University Hospital between September 2020 and March 2021. All patients underwent fluorescein angiography and spectral-domain optical coherence tomography (SD-OCT) using the Heidelberg Spectralis (Heidelberg Retinal Angiography; Heidelberg Engineering, Heidelberg, Germany, Software version 6.9) platform. These exams were performed between five and fifteen days from the clinical diagnosis. Data regarding best-corrected visual acuity (BCVA), refraction, age, sex, medical history, and slit-lamp examination were also recorded. Visual acuity was converted in the logarithm of the minimal angle of resolution (logMAR) for statistical purposes. We excluded cases with spherical equivalent greater than ±6 diopters and cylinder greater than ±2 diopters, previous laser photocoagulation or anti-vascular endothelial growth factor therapy in any eye, history of trauma or intraocular surgery except for cataract extraction at least 180 days before enrollment, other retinal disorders that could interfere with the measurement including age-related macular degeneration, diabetic retinopathy, central serous chorioretinopathy, any history of uveitis.

OCT images were obtained with the enhanced deep image (EDI) protocol acquiring 20 equally spaced OCT B-scan sections in a 20° × 20° horizontal raster pattern. Choroidal thickness (CT) was measured in the subfoveal region using the built-in caliper of the software of the instrument at a single point below the fovea extending from the bottom of the hyperreflective layer corresponding to Bruch’s membrane to the hyperreflective layer at the sclerochoroidal border. Central macular thickness (CMT) was recorded for all patients using the embedded tool.

We classified the images into 3 categories, depending on the pattern of macular edema: no macular edema (nME), cystoid macular edema (CME), cystoid macular edema with serous retinal detachment (mixed).

### 2.1. Image Binarization

**Figure 1:**
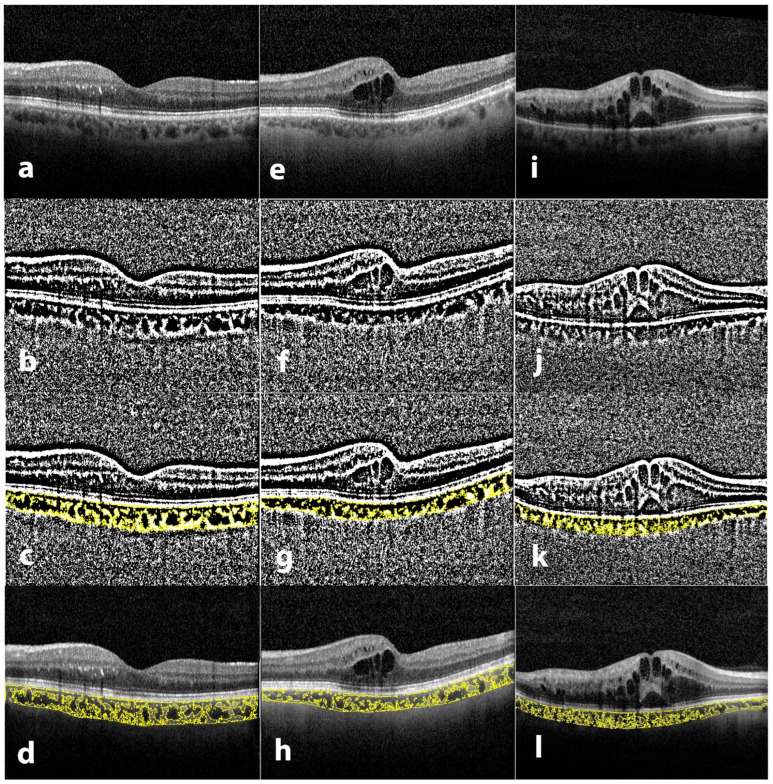
Binarization and identification of the luminal and stromal areas of the choroid. Spectral-domain optical coherence tomography (SD-OCT) acquired using enhanced deep image (EDI) mode. (**a**,**e**,**i**) Original subfoveal scan; (**b**,**f**,**j**) The image was downgraded to 8-bit and Niblack auto local threshold was applied; (**c**,**g**,**k**) Color threshold was used to select luminal area; (**d**,**h**,**l**) Overlay of region of interest on the original image. Depending on the pattern of macular edema, eyes were classified into 3 categories: no macular edema (**a**), cystoid macular edema (**e**), cystoid macular edema with serous retinal detachment (**i**).

The same full-length scan used for CT measurement was utilized for binarization employing the open-source software ImageJ (version 1.52; National Institutes of Health, USA, http://imagej.nih.gov/ij, accessed on 9 June 2022). The polygon tool was used to select the TCA. The selection was added to the region of interest (ROI) manager. The image was then downgraded to 8-bit and adjusted with Niblack auto local threshold. Color threshold was used to select the LA which was added to the ROI manager. CVI was calculated as the proportion of LA to TCA. Stromal area (SA) was calculated by subtracting LA from TCA (Figure 1).

### 2.2. Statistical Analysis

Statistical analysis was performed using the SPSS software version 20.0 for Windows (SPSS Inc., Chicago, IL, USA). The normality of distribution of data was assessed using Kolmogorov–Smirnov and Shapiro–Wilk tests. Differences in SFCT, CMT, TCA, SA, LA and CVI were assessed with a two-side independent sample *t*-test. Fisher’s exact test was applied for categorical variables. Analysis of variance test and Pearson χ^2^ test were used comparing demographics and clinical features between different types of macular edema. *p* values < 0.05 were considered statistically significant.

## 3. Results

Seventy-six consecutive patients affected by BRVO or CRVO were screened. Among these, 29 were excluded due to poor image quality, concomitant retinal diseases, or recent surgery; the remaining 47 subjects were included in the study. Patient demographics and clinical characteristics are displayed in Table 1.

The eyes of subjects with BRVO or CRVO did not significantly differ regardless of gender and laterality distribution, mean age, concomitant systemic diseases, ischemic pattern, and type of macular edema. BCVA was significantly lower in patients with CRVO than in those with BRVO (0.811 ± 0.30 (20/126) and 0.565 ± 0.39 (20/73), respectively, *p* = 0.041, independent sample *t*-test). The type of macular edema was nME in 9 cases, CME in 23 cases and mixed in 15 cases (Table 2).

Mean age was significantly lower in eyes with nME (*p* = 0.025, analysis of variance test). No difference was observed between groups in gender, laterality distribution and BCVA.

Comparing eyes with RVO with their unaffected fellow eyes, we observed no significant difference in subfoveal choroidal thickness and SA. In contrast, eyes with RVO had greater values of CMT, TCA, LA and CVI (Table 3).

Table 4 displays OCT parameters according to the type of macular edema. When compared with controls, eyes with nME had no significant differences in SFCT, CMT, TCA, LA, SA and CVI. Eyes with CME presented a significant increase in SFCT, CMT, TCA, LA and CVI. Finally, eyes with mixed macular edema had greater CMT and CVI than contralateral eyes.

Considering OCT parameters according to the type of retinal occlusion, eyes with CRVO and BRVO did not significantly differ except for CMT (Table 5).

We also compared OCT parameters in eyes with CRVO; no significant difference was found between eyes with ischemic and non-ischemic subtype. 

## 4. Discussion

In this cross-sectional study, we evaluated choroidal vascularity changes in patients affected by retinal vein occlusion, both central and branch, using their unaffected fellow eyes as control. We further aimed to investigate if there was any difference in choroidal response among central and branch RVO and if there was a relationship between choroidal vascularity and the type of macular edema. 

Taken together, we observed an increase in CVI as well as TCA and LA in affected eyes compared to controls. Comparing eyes with central and branch RVO, there was no difference in any of the considered OCT parameters except for CMT. Similar findings were reported by Tang and colleagues [16] meaning that the vascularity choroidal changes may be equal regardless of the type of occlusion. A recent study found that CVI was significantly lower in patients with RVO than in fellow eyes. However, the measurements were performed at least 1 month after an anti-vascular endothelial growth factor (VEGF) injection or steroid (dexamethasone) implant [17]. Consequently, the lower values of CVI could have been influenced by the effect of the drugs and the reduction of macular edema, although the effect of steroids on choroid is still controversial. Similarly, it has been reported that choroidal thickness reduced after anti-VEGF or intravitreal steroid therapy [8,18,19].

In our study, no differences in subfoveal choroidal thickness arose between the study and contralateral eyes. Recent research evaluating choroidal thickness change in patients with RVO reported discordant results. Some authors demonstrated a greater choroidal thickness in eyes with RVO if compared with the unaffected fellow eyes [7,16,20]. In contrast, others [8,9] showed no differences in choroidal thickness between the affected and contralateral eyes. Even after treatment, changes in choroidal thickness were different [8]. Some studies reported a significant SFCT change after intravitreal injections [7], whereas other studies reported that SFCT did not decrease after treatment [21].

Furthermore, SFCT did not change in functional non-responders after 3 monthly anti-VEGF therapy [5,22]. Although the reasons for this difference were not completely understood, a possible explanation may be attributed to several factors such as type of RVO, central or branch, RVO phase (acute or longstanding), type of macular edema, age, axial length, gender, anterior chamber depth, and lens thickness. The CVI has the advantage of overlooking the limitations related to the use of CT since it is not influenced by the aforementioned variables.

Comparing eyes with CRVO and BRVO, we found that CMT was greater in eyes with CRVO. Previous studies [23,24] demonstrated that SFCT was thicker in eyes with CRVO than in those with BRVO. It had been observed that CRVO eyes had a higher ischemic index and VEGF level compared with BRVO eyes [25]. Increased VEGF may be the main cause of increased choroidal thickness inducing vascular hyperpermeability and dilated vessels in the choroidal layer [26,27]. It may also explain the increased CMT observed in eyes with CRVO.

We stratified our results according to the type of macular edema. There was no difference between subtypes of macular oedema regardless of age, gender, laterality and BCVA. If compared with controls, eyes with CME had higher values of SFCT, CMT, TCA, LA and CVI. Increased CMT and CVI have been observed also in eyes with a mixed pattern of macular edema (intraretinal and subfoveal). In contrast, we noticed no difference in SFCT, CMT, TCA, LA, SA and CVI in the nME group.

We hypothesize that eyes with CME and mixed macular edema could have a greater inflammatory response secondary to RVO comparing with nME eyes. It is known that, when RVO occurs, the choriocapillaris increases its permeability under the influence of soluble VEGF and other inflammatory mediators [28]. VEGF induces choroidal vascular hyperpermeability and, subsequently, choroidal thickening [7]. Moreover, choroidal thickening is also due to nitric oxide production, triggered by VEGF expression [28]. It is possible that in eyes with nME, this inflammatory response was absent or reduced so even choroidal thickening does not occur. To confirm this theory, our results showed an increased CVI only in eyes in which macular edema had developed.

Although CVI has been extensively discussed and globally recognized as a reliable and promising tool for the study of choroidal vascularity, there are some limitations in its routine use by regular ophthalmologists in their daily activity. The EDI-OCT images should be binarized which requires a certain familiarity with the software. However, CVI could provide interesting information as it could be used as a prognostic parameter to be taken into consideration in the proper management of eyes with RVO.

This study suffers from certain limitations including the retrospective design and the small sample sizes in the subgroups. Additionally, we measured only a single scan going through the fovea; a volume scan over the macular area could provide more comprehensive information.

## 5. Conclusions

In conclusion, our results support a dilatation of the choroidal vessels in eyes with RVO. Choroidal vascularity, assessed by the CVI, varied according to the pattern of macular edema (nME, CME, mixed) and was independent of the type of RVO (branch or central).

## Figures and Tables

**Table 1:** Patient demographics and clinical characteristics in subjects with recent-onset retinal vein occlusion.

	RVO	BRVO	CRVO	*p* Value
No. of eyes	47	36	11	
Gender (male/female)	26/21	20/16	6/5	0.610 ^†^
Eye (right/left)	28/19	23/13	5/6	0.312 ^†^
Age (years, mean ± SD)	72.40 ± 11.64	73.03 ± 14.45	64.57 ± 8.61	0.431 *
Systemic disease				
Hypertension	28	22	6	0.697 ^†^
Diabetes mellitus	6	4	2	0.538 ^†^
Glaucoma	9	7	2	0.925 ^†^
Ischemic/non ischemic	21/26	14/22	7/4	0.181 ^†^
Type of macular oedema				
nME	9	9	0	n/a
CME	23	18	5	0.791 ^†^
Mixed	15	9	6	0.065 ^†^
BCVA	0.623 ± 0.38	0.565 ± 0.39	0.811 ± 0.30	0.041 *

RVO = retinal vein occlusion; BRVO = branch retinal vein occlusion; CRVO = central retinal vein occlusion; nME = no macular oedema; CME = cystoid macular oedema; Mixed = cystoid macular oedema + serous retinal detachment; BCVA = best corrected visual acuity. * BRVO compared to CRVO, independent-sample *t*-test. ^†^ Fisher’s exact test.

**Table 2:** Demographics and clinical features according to type of macular oedema in subjects with recent-onset central or branch retinal vein occlusion.

	Macular Oedema Group				
	nME Group (*n* = 9)	CME Group (*n* = 23)	Mixed Group (*n* = 15)	Control Group (*n* = 47)	*p* Value
Age (mean ± SD)	63.11 ± 14.61	74.74 ± 11.45	74.40 ± 6.96	72.40 ± 11.64	0.025 *
Gender(male/female)	5/4	14/9	7/8	26/21	0.690 ^†^
Eye (right/left)	5/4	16/7	7/8	28/19	0.359 ^†^
BCVA (logMAR)	0.2 ± 0.16	0.63 ± 0.32	0.87 ± 0.35	0.62 ± 0.38	0.142 *
Type (CRVO/BRVO)	0/9	3/20	8/7	11/36	0.003 ^†^

BCVA = best-corrected visual acuity; nME = no macular oedema; CME = cystoid macular oedema; Mixed = cystoid macular oedema + serous retinal detachment; CRVO = central retinal vain occlusion; BRVO = branch retinal vein occlusion. * Analysis of variance test. ^†^ Pearson χ^2^ test.

**Table 3:** Optical coherence tomography parameters in patients with recent-onset retinal vein occlusion.

Parameter	Study Eye	Fellow Eye	p *
Subfoveal choroidal thickness (µm)	200.87 ± 56.24	176.83 ± 44.89	0.24
Central macular thickness (µm)	505.49 ± 218.28	276.93 ± 28.45	**<0.001**
Total choroidal area (mm^2^)	0.44 ± 0.13	0.39 ± 0.11	**0.046**
Luminal area (mm^2^)	0.17 ± 0.05	0.13 ± 0.04	**0.002**
Stromal area (mm^2^)	0.27 ± 0.08	0.25 ± 0.07	0.218
Choroidal vascularity index (%)	38.46 ± 3.89	35.64 ± 3.24	**<0.001**

* Independent-sample *t*-test. Significant *p* values are in bold.

**Table 4:** Optical coherence tomography parameters according to type of macular oedema secondary to retinal vein occlusion.

Parameter/Group	nME	Control	*p* Value *	CME	Control	*p* Value ^†^	Mixed	Control	*p* Value ^ǂ^
SFCT (µm)	216.22 ± 80.63	205.89 ± 44.19	0.740	208.91 ± 51.61	174.39 ± 43.94	**0.019**	179.33 ± 163.13	163.13 ± 41.45	0.295
CMT (µm)	332.44 ± 87.44	290.66 ± 38.09	0.207	464.82 ± 166.19	270.39 ± 24.98	**<0.001**	671.66 ± 239.22	278.73 ± 25.59	**<0.001**
TCA (mm^2^)	0.51 ± 0.17	0.47 ± 0.14	0.634	0.44 ± 0.13	0.37 ± 0.10	**0.039**	0.39 ± 0.92	0.37 ± 0.89	0.426
LA (mm^2^)	0.19 ± 0.06	0.16 ± 0.05	0.380	0.17 ± 0.05	0.13 ± 0.03	**0.006**	0.15 ± 0.04	0.13 ± 0.04	0.111
SA (mm^2^)	0.32 ± 0.11	0.30 ± 0.10	0.802	0.27 ± 0.08	0.23 ± 0.06	0.115	0.24 ± 0.05	0.23 ± 0.05	0.981
CVI (%)	37.53 ± 3.33	35.50 ± 3.55	0.230	38.28 ± 3.55	36.10 ± 3.12	**0.032**	39.29 ± 4.72	35.01 ± 3.35	**0.008**

SFCT = subfoveal choroidal thickness; CMT = central macular thickness; TCA = total choroidal area; LA = luminal area; SA = stromal area; CVI = choroidal vascularity index; nME = no macular oedema; CME = cystoid macular oedema; Mixed = cystoid macular oedema + serous retinal detachment. * nME group compared to control group, independent-sample *t* test. ^†^ CME group compared to control group, independent-sample *t* test. ^ǂ^ Mixed group compared to control group, independent-sample *t* test. Significant *p* values are in bold.

**Table 5:** Optical coherence tomography parameters according to type of retinal vein occlusion.

Parameter/Group	BRVO	CRVO	*p* Value *
SFCT (µm)	199.08 ± 58.68	206.76 ± 49.46	0.673
CMT (µm)	443.28 ± 167.59	709.09 ± 247.66	**0.005**
TCA (mm^2^)	0.45 ± 0.14	0.39 ± 0.09	0.090
LA (mm^2^)	0.17 ± 0.05	0.14 ± 0.04	0.091
SA (mm^2^)	0.28 ± 0.09	0.24 ± 0.05	0.115
CVI (%)	38.83 ± 3.87	37.22 ± 3.88	0.245

CRVO = central retinal vein occlusion; BRVO = branch retinal vein occlusion; SFCT = subfoveal choroidal thickness; CMT = central macular thickness; TCA = total choroidal area; LA = luminal area; SA = stromal area; CVI = choroidal vascularity index. * Independent-sample *t* test. Significant *p* values are in bold.

## Data Availability

Data supporting reported results can be found in Supplementary Materials, Table S1.

## Supplementary Materials

The following supporting information can be downloaded at: https://www.mdpi.com/article/10.3390/jcm11164756/s1, Table S1: data set.
